# Calculation of π and Classification of Self-avoiding Lattices via DNA Configuration

**DOI:** 10.1038/s41598-019-38699-0

**Published:** 2019-02-19

**Authors:** Anshula Tandon, Seungjae Kim, Yongwoo Song, Hyunjae Cho, Saima Bashar, Jihoon Shin, Tai Hwan Ha, Sung Ha Park

**Affiliations:** 1 Department of Physics and Sungkyunkwan Advanced Institute of Nanotechnology (SAINT), Sungkyunkwan University, Suwon, 16419 Korea; 2 Hazards Monitoring BNT Research Center, Korea Research Institute of Bioscience and Biotechnology (KRIBB), Daejeon, 34141 Korea; 3 Department of Nanobiotechnology, KRIBB School of Biotechnology, Korea University of Science and Technology (UST), Daejeon, 34113 Korea

## Abstract

Numerical simulation (*e*.*g*. Monte Carlo simulation) is an efficient computational algorithm establishing an integral part in science to understand complex physical and biological phenomena related with stochastic problems. Aside from the typical numerical simulation applications, studies calculating numerical constants in mathematics, and estimation of growth behavior via a non-conventional self-assembly in connection with DNA nanotechnology, open a novel perspective to DNA related to computational physics. Here, a method to calculate the numerical value of π, and way to evaluate possible paths of self-avoiding walk with the aid of Monte Carlo simulation, are addressed. Additionally, experimentally obtained variation of the π as functions of DNA concentration and the total number of trials, and the behaviour of self-avoiding random DNA lattice growth evaluated through number of growth steps, are discussed. From observing experimental calculations of π (π_exp_) obtained by double crossover DNA lattices and DNA rings, fluctuation of π_exp_ tends to decrease as either DNA concentration or the number of trials increases. Based upon experimental data of self-avoiding random lattices grown by the three-point star DNA motifs, various lattice configurations are examined and analyzed. This new kind of study inculcates a novel perspective for DNA nanostructures related to computational physics and provides clues to solve analytically intractable problems.

## Introduction

A multitude of analytically intractable problems in various disciplines are addressed by performing numerical simulations that employ a computational model of a system to describe its complex behaviour over a time period by incorporating given variables. One such commonly used model is Monte Carlo (MC) simulation^1^ that refers to an effective computational algorithm adopted to perform an underlying stochastic and random sampling experiment on a computer to calculate various outcomes. MC simulation is used in science and engineering to understand complex physical phenomena, generate useful mathematical functions, and predict complicated algorithmic processes. Interestingly, the MC method has also been effectively used to understand complex biological process mechanisms such as the biological self-assembly behaviour, biomolecule dynamics, and the interaction between biomolecules and nanomaterials^2–23^.

Among typical MC simulation applications, there are two interesting ones; calculating π (one of most important mathematical constants defined as the ratio of a circle’s circumference to its diameter), and interpreting a self-avoiding walk (an abstract model describing the behaviour of chain like entities where no two points can occupy the same place)^24^. Several approaches have been adapted to calculate π, among which the famously used one is Buffon’s needle approach^25^. The MC method is also used to enumerate the characteristics of the self-avoiding walk, to interpret the possibility to estimate proper paths.

The fabrication of various dimensional DNA nanostructures is well established due to the programmability of DNA base sequences and the stability of DNA molecules. Although these artificially designed DNA nanostructures find various applications as physical, chemical, or biomedical devices and sensors^26–32^, calculating mathematical constants and incorporating abstract modeling via DNA nanostructures are rarely discussed.

Here, we develop ways to calculate π and evaluate the applicable number of self-avoiding walk paths with the aid of the computational simulation. In addition, we experimentally demonstrate the calculation of π and evaluate applicable self-avoiding walk paths with two different DNA nanostructures (double crossover DNA lattices and DNA rings) and self-avoiding random DNA lattices (constructed by a three-point star DNA motif having a blunt-end), respectively. Finally, we analyze the trend of numerical π variations controlled by DNA concentration, the total number of trials, and the characteristic growth behaviour of self-avoiding random DNA lattices evaluated through the total number of growth steps for the self-avoiding walk path.

## Results

### Calculation of π value

**Figure 1:**
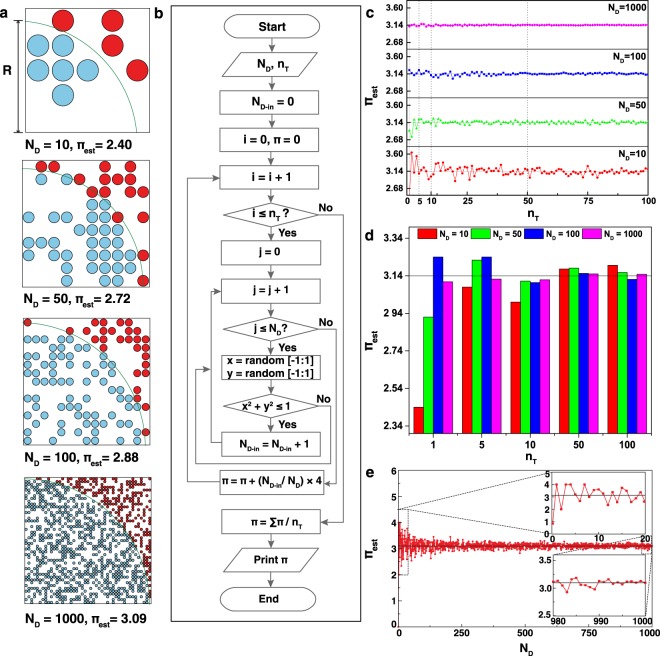
Calculation of π using Monte Carlo simulation. (**a**) The representative schematics for π calculation with a different number of dots in a square. Calculated numerical value of π (=π_est_, where est stands for estimation) is defined as (N_D-in_/N_D_) × 4, where N_D-in_ and N_D_ stand for the number of dots inside a quadrant of a circle (with a radius of R) and the total number of dots in a square (with a length of R). By definition, π_est_ with four different N_D_, *i*.*e*. 10, 50, 100, and 1000 are calculated to be 2.40 (=6/10 × 4), 2.72, 2.88 and 3.09 respectively, showing that roughly larger N_D_ gives a more accurate known value of π (π_known_ ≈ 3.14). (**b**) A flow chart depicting algorithmic steps to obtain π_est_ with various N_D_ and total number of trials (n_T_). (**c**) π_est_ as a function of n_T_ at a given N_D_ (*e*.*g*. 10, 50, 100, or 1000). In general, π_est_ approaches to π_known_ with the increasing n_T_ at relatively larger N_D_ values, as expected. (**d**) π_est_ as a function of N_D_ at a fixed n_T_ (*e*.*g*. 1, 5, 10, 50, or 100 marked as a dotted line in (**c**). From observation, π_est_ approaches to π_known_ with the increasing N_D_ at relatively smaller n_T_ but π_est_ is roughly independent with N_D_ at relatively larger n_T_. (**e**) A representative graph of π_est_ as a function of N_D_. As N_D_ is increased, fluctuation of π_est_ from π_known_ tends to decrease. Insets show tendencies of fluctuation of π_est_ in the two different ranges of N_D_.

The representative schematics for π calculation with a different number of dots in a square having a quadrant of a circle are shown in Fig. 1a. For acquiring an calculated numerical value of π (=π_est_, where est stands for estimation), a random event needs to be considered which can be defined as drawing uniformly distributed dots (like throwing darts randomly at a board) over a square bounding box within the region whose area is to be determined. By considering a quadrant of a circle with a radius R bounded by a square with a length R, the ratio of the quadrant area to the square area is approximately equal to the ratio of the total number of dots falling inside the quadrant (N_D-in_, marked as blue) to the total number of dots inside the square (N_D_) due to the uniformly distributed dots within the square. Therefore, π_est_ can be defined as (N_D-in_/N_D_) × 4. By definition, representative π_est_ with four different N_D_ (*i*.*e*. 10, 50, 100, and 1000) are calculated to be 2.40 (=6/10 × 4), 2.72, 2.88 and 3.09 respectively. This shows that a roughly larger N_D_ gives a relatively more accurate known value of π (π_known_ ≈ 3.14). Consequently, the magnitude (*i*.*e*. 0.060 = |3.2–3.14|, 0.020, 0.019, and 0.001) of the deviation of π_est_ from π_known_ (∆π_exp_ = |π_est_ − π_known_|) will be smaller as N_D_ (10, 50, 100, and 1000) increases at a given optimum N_D-in_ (*i*.*e*. 8, 39, 79, and 785, which provides the most accurate π_est_ compared to π_known_ at a given N_D_).

Figure 1b shows a flowchart representing algorithmic steps in order to obtain π_est_ as a function of either N_D,_ or the total number of trials (n_T_). By assigning an initial input of N_D_ with the unit-step increment of j, dots are randomly sampled in a square. Then, N_D-in_ are counted until j reaches to N_D_ followed by evaluation of π_est_ calculated as (N_D-in_/N_D_) × 4. Similarly, when the unit-step increment of i reaches an initial input of n_T_, summed π_est_ is divided by n_T_ to get the average π_est_.

By using the algorithm for π_est_, numerical values of the π_est_ as functions of N_D_ and n_T_ can be obtained and analyzed. π_est_ as a function of n_T_ at four different N_D_ values (*i*.*e*. 10, 50, 100, and 1000) are obtained, which approaches π_known_ with the increasing n_T_ at any given N_D_ values, as expected (Fig. 1c). The π_est_ with varying N_D_ at a fixed n_T_ (*e*.*g*. 1, 5, 10, 50, or 100 marked as a dotted line in Fig. 1c) are extracted in order to evaluate the trend of π_est_ as a function of N_D_ which shows that π_est_ heavily relies on N_D_ at relatively smaller n_T_ but it is roughly independent of N_D_ at larger n_T_ (Fig. 1d). A representative graph of π_est_ as a function of N_D_ is shown in Fig. 1e. As N_D_ is increased, the fluctuation of π_est_ from π_known_ tends to decrease. Insets show the fluctuation tendency of π_est_ in the two different ranges of N_D_ (*i*.*e*. between 0~20 and 980~1000), which clearly shows that fluctuation of π_est_ from π_known_ tends to decrease with the increase in N_D_, as expected. In addition, the differentiation of π_est_ per unit number of dots (=Δπ_est_ /ΔN_D_) as a function of N_D_ is shown in Supplementary Fig. 1. Differences in the π_est_ per unit number of dots tend to decrease with the increase in N_D_ because π_est_ at a relatively larger N_D_ has a greater chance to give an accurate value of π.

### Experimental observation of π using DNA nanostructures

**Figure 2:**
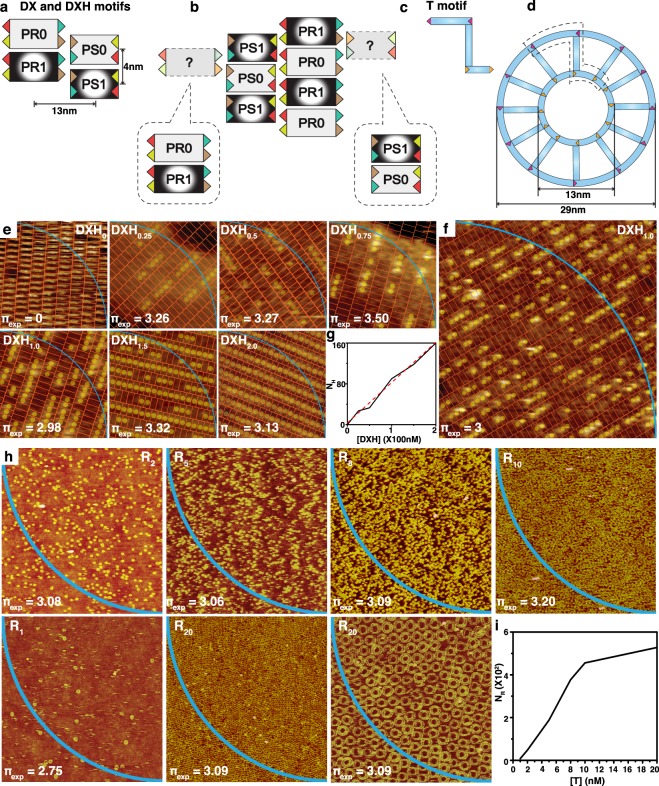
Experimental observation of π using DNA nanostructure configuration. (**a**,**b)** Cartoon representations of two sets ‒ PR and PS ‒ of DNA double-crossover (DX) motifs and corresponding DX lattice formed by complementary colour-coded sticky ends. Each set has two DX motifs, without and with hairpins marked as PR(S)0 and PR(S)1, respectively. Hairpins with a length of 3.5 nm protruding up and down on a DX motif called as a DXH. DX and DXH motifs having identical sets of sticky ends in each set can hybridize to form a DX lattice (two exemplified binding sites are indicated by question marks) with the equal probability of binding. (**c**,**d)** Schematics of unit building block (called as a T motif) and a DNA ring made of T motifs. The complementary-counterparts are colour-coded with the same colours. (**e**,**f)** AFM images of DX lattices with different concentrations of DXH (0, 25, 50, 100, 150 and 200 nM represented as DXH_0_, DXH_0.25_, DXH_0.5_, DXH_1.0_, DXH_1.5_ and DXH_2.0_ respectively) annealed in free solution. An arc (shown in blue) in each image is drawn representing first quadrant in a circle. Experimental observation of π through images (π_exp_) can be obtained by (N_H-in_/N_H_) × 4, where N_H-in_ and N_H_ represent the number of hairpins inside a quadrant of a circle and total number of hairpins in an image. A scan size of all images in (**e**,**f**) is 100 × 100 nm^2^ (200 × 200 nm^2^). (**g**) A graph of concentration of DXH ([DXH])-dependent N_H_ analyzed by AFM images with the scan size of 100 × 100 nm^2^. Theoretical and experimental N_H_ are plotted as red-dotted and black-solid lines, respectively. (**h**) AFM images of DNA rings with different concentrations of a T motif (2, 5, 8, 10 nM with the scan size of 3 × 3 μm^2^, 1 and 20 nM with 2 × 2 μm^2^, and 20 nM with 600 × 600 nm^2^ indicated as R_2_, R_5_, R_8_, R_10_, R_1_, R_20_ and R_20_, respectively) annealed through a mica-assisted growth method. Arcs are drawn in third quadrants and corresponding π_exp_ (measured by (N_R-in_/N_R_) × 4, where N_R-in_ and N_R_ represent number of rings inside a quadrant and the total number of rings in an image) are shown in images. (**i)** A plot of N_R_ as a function of [T] analyzed by AFM images with scan size of 1 × 1 μm^2^.

Experimental observation of π (π_exp_) is demonstrated by constructing two types of DNA nanostructures, *i*.*e*. double crossover (DX) DNA lattices^33,34^ and DNA rings^35–37^ (Fig. 2). Two sets of DX DNA motifs (*i*.*e*. PR and PS) are designed for construction of DX DNA lattices. Here, P stands for Pi (π) and R/S indicate opposite helical directionalities of the duplexes within the motifs (See Supplementary Fig. 2, Supplementary Tables 1 and 2). Each set has two DX motifs, without and with hairpins marked as PR(S)0 and PR(S)1, respectively (Fig. 2a). A DX motif having hairpins ~3.5 nm long protruding up and down is called DXH (*i*.*e*. PR1 and PS1). DX and DXH motifs, having identical sets of sticky ends in each set with the equal probability of binding (two exemplified binding sites are indicated by question marks in Fig. 2b), can hybridize to form a DX lattice with the aid of complementary colour-coded and shape-coded sticky ends. In addition, DNA rings comprised of T motifs (non-crossover based DNA motifs having three double-stranded domains connected through single strands. See Fig. 2c, Supplementary Fig. 3, and Supplementary Tables 3 and 4) are fabricated in order to obtain π_exp_. A ring with inner and outer diameters of 13 nm and 29 nm is constituted through the complementary base-pairs of the sticky ends in T motifs (Fig. 2d).

Representative structural configurations of DX DNA lattices and DNA rings are shown in Fig. 2e,h, respectively. Atomic force microscope (AFM) images of DX lattices with different concentrations of DXH (0, 25, 50, 100, 150, and 200 nM symbolized as DXH_0_, DXH_0.25_, DXH_0.5_, DXH_1.0_, DXH_1.5_, and DXH_2.0_, respectively) were annealed in free solution. An arc (shown in blue) in each image is drawn representing the first quadrant in a circle. π_exp_ (0.00, 3.26, 3.27, 3.50, 2.98, 3.32, and 3.13) through images in Fig. 2e are obtained by (N_H-in_/N_H_) × 4, where N_H-in_ and N_H_ represent the number of hairpins inside a quadrant of a circle and total number of hairpins in an image. Four circle quadrants can be assigned on a given image, which provide specific π_exp_. DXH concentration ([DXH])-dependent N_H_ values (roughly linearly dependent) obtained by theoretical calculation and analyzed by AFM images are displayed in Fig. 2g. Similarly, AFM images of DNA rings with different concentrations of a T motif (1, 2, 5, 8, 10, and 20 nM indicated as R_1_, R_2_, R_5_, R_8_, R_10_, and R_20_, respectively) were annealed through a mica-assisted growth method^38–40^ (Fig. 2h). Arcs are drawn in third quadrants and corresponding π_exp_ (ranging between 2.75 and 3.20 measured by (N_R-in_/N_R_) × 4, where N_R-in_ and N_R_ indicate number of rings inside a quadrant and total number of rings in an image) are shown in the images. Lastly, a plot of N_R_ as a function of [T] (roughly sigmoidal) analyzed by AFM images is shown in Fig. 2i.

### Analysis of π using experimental observation

**Figure 3:**
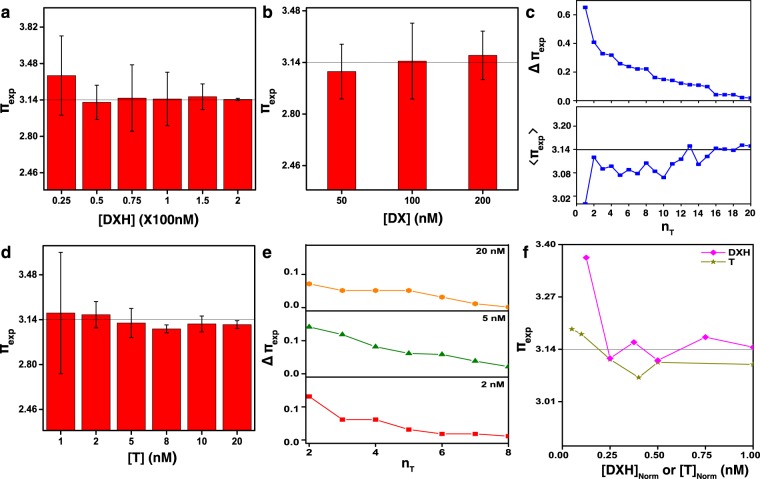
The analysis of experimentally obtained π (π_exp_) controlled by DNA concentrations ([DNA]) and the number of trials (n_T_). (**a**) A histogram plot of π_exp_ as a function of concentrations of a DX motif with the hairpin ([DXH]) at a fixed concentration of each motif set ([DX_PR_] = [DX_PS_] = 100 nM). Here, [DXH] is defined as the concentration sum of DXHs in each set of motif (=[DX_PR1_] + [DX_PS1_]). For instance, 50 nM of [DXH] means 50 nM of [DX_PR1_] + [DX_PS1_] with 150 nM of [DX_PR0_] + [DX_PS0_]. Average π_exp_ is obtained from more than four data sets at a given [DXH]. The magnitude of the deviation of π_exp_ from π_known_ (∆π_exp_ = |π_exp_ − π_known_|) is almost constant above 50 nM of [DXH]. (**b**) A plot of π_exp_ as a function of [DX] ( = [DX_PR_] or [DX_PS_] with the condition of [DX_PR_] = [DX_PS_]) with the equal amount of DX motifs without and with hairpins ([DX_PR0_] = [DX_PR1_] and [DX_PS0_] = [DX_PS1_]). As an example, 100 nM of [DX] indicates [DX_PR_] = [DX_PS_] = 100 nM having 50 nM of each [DX_PS0_] and [DX_PS1_] as well as 50 nM each of [DX_PR0_] and [DX_PR1_]. By observation, 100 nM of [DX] gives more accurate π_exp_ (3.15) than 50 (3.08) or 200 nM (3.19) of [DX]. (**c**) Plots of the deviation of π_exp_ (∆π_exp_) (arranged in a descending order) and average π_exp_ (〈π_exp_〉 = $\sum _{n=1}^{{n}_{T}}{\pi }_{exp,n}/n\,$) as a function of n_T_. Here, 100 nM of [DX] (=[DX_PR_] = [DX_PS_]) with 50 nM of each [DX_PR(S)0_] and [DX_PR(S)1_] are used. (**d)** A histogram plot of π_exp_ as a function of [T]. Accidentally, ∆π_exp_ are roughly independent of [T]. (**e)** Plots of ∆π_exp_ arranged in a descending order as a function of n_T_ at 2, 5, and 20 nM of [T]. Although 20 nM of [T] shows slightly less ∆π_exp_ than other [T], roughly 〈π_exp_〉 are independent with [T] which is in agreement with (**d**.**f)** A graph of π_exp_ against normalized [DXH] ([DXH]_Norm_ = [DXH]/[DXH]_200_) and normalized [T] ([T]_Norm_ = [T]/[T]_20_). It shows comparison of π_exp_ with the two different DNA nanostructure configurations (*i*.*e*. lattices and rings).

The analysis of π_exp_ controlled by [DX] and n_T_ are conducted and results are displayed in Fig. 3. The histogram in Fig. 3a shows an average of π_exp_ (〈π_exp_〉 obtained from more than four data sets at a given [DXH]) as a function of [DXH] (the concentration sum of DXHs in each set of motif, [DX_PR1_] + [DX_PS1_]) *i*.*e*. 25, 50, 75, 100, 150, and 200 nM at a fixed [DX_PR_] and [DX_PS_] of 100 nM. For example, 150 nM of [DXH] indicates 150 nM of [DX_PR1_] + [DX_PS1_] with 50 nM of [DX_PR0_] + [DX_PS0_]. Although the standard deviation of an error bar generally decreases as [DXH] increases, the magnitude of the deviation of π_exp_ from π_known_ (∆π_exp_ ≡ |π_exp_ - π_known_|) is almost constant above 50 nM of [DXH]. A plot of π_exp_ as a function of [DX] ( = [DX_PR(S)_] with [DX_PR(S)0_] = [DX_PR(S)1_]) is shown in Fig. 3b. By observation, 100 nM of [DX] gives a more accurate π_exp_ (∆π_exp_ of ~0.009) than 50 (~0.062) or 200 nM (~0.049) of [DX]. Figure 3c displays ∆π_exp_ (arranged in a descending order) and 〈π_exp_〉 (defined as $\sum _{n=1}^{{n}_{T}}{\pi }_{exp,n}/n)$ as a function of n_T_ at a fixed [DX] of 100 nM which provide the general behaviour of π_exp_ approaching π_known_ with increasing n_T_, as expected.

Similarly, π_exp_ and ∆π_exp_ as functions of [T] and n_T_ analysed from DNA rings are discussed. As observed from the bar graph of π_exp_ in Fig. 3d, the standard deviation of an error bar roughly decreases as [T] increases and ∆π_exp_ is approximately independent with [T], which might be due to the uniform distribution of the DNA rings on a given substrate. Curves of ∆π_exp_ arranged in a descending order as a function of n_T_ at 2, 5, and 20 nM of [T] are displayed in Fig. 3e. Although 20 nM of [T] shows slightly less ∆π_exp_ than other [T], roughly 〈π_exp_〉 are independent from [T] which is in good agreement with Fig. 3d. π_exp_ against normalized [DXH] ([DXH]_Norm_ = [DXH]/[DXH]_200_) and normalized [T] ([T]_Norm_ = [T]/[T]_20_) are shown in Fig. 3f in order to compare π_exp_ with respect to either largest [DXH] or [T], as well as to understand comparison of π_exp_ with the two different DNA nanostructure configurations, *i*.*e*. DNA lattices and DNA rings.

### Self-avoiding random lattice growth

**Figure 4:**
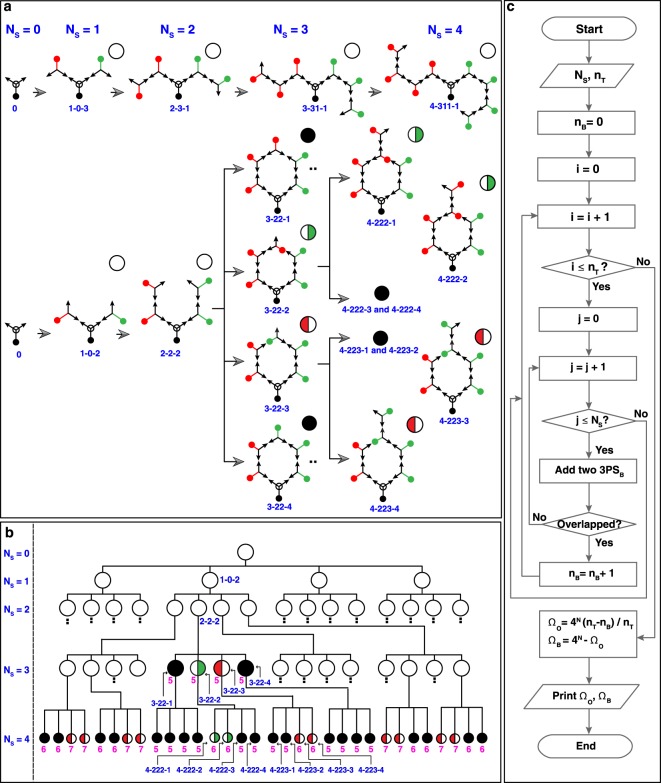
Lattice configuration of self-avoiding random lattice growth demonstrated with the three-point star motif having a blunt-end. **(a)** Schematic representations of lattices constructed by a three-point star motif having a blunt-end (3PS_B_). A blunt-end is marked with either a black (served as a seed), a red (grown to the left), or a green dot (grown to the right). Lattice configurations are named as (step number, N_S_)-(configuration number from the previous step)-(configuration number at the present step). For instance, 2-3-1 represents 1^st^ configuration of 2^nd^ step obtained from 3^rd^ configuration in 1^st^ step. There are two types of available lattice configurations,.*i*.*e*. open and blocked (half- and full-blocked indicated by half- and fully-filled circles, respectively) lattice configurations. (**b)** A pedigree lattice configuration chart of self-avoiding random growth. 32 blocked lattice configurations ‒ 10 (2) half-blocked happened on the left (right) side of the lattices, and 20 full-blocked configurations ‒ out of 256 available configurations $({{\rm{\Omega }}}_{{{\rm{N}}}_{{\rm{S}}}}={4}^{{{\rm{N}}}_{{\rm{s}}}})$ after 4^th^ step (N_S_ = 4) of lattice growth are shown. Total number of the 3PS_B_ (excluding a seed 3PS_B_) participated in that configuration is indicated by magenta. (**c)** A flow chart depicting algorithmic steps to obtain the total numbers of open (Ω_O_) and blocked (Ω_B_) lattice configurations at a given N_S_.

A self-avoiding random walk path (called a lattice configuration) constructed by a unit building block is demonstrated via MC simulation in order to understand the feasibility to predict proper paths. A self-avoiding random lattice has a growth path on a lattice configuration that does not visit the same place more than once. Schematics of various lattice configurations constructed by a three-point star motif having single blunt-end (3PS_B_) are represented in Fig. 4a. A blunt-end in a 3PS_B_, which is introduced to generate asymmetric self-avoiding random lattices, is marked with a black (serves as a seed), a red (grown to the left), or a green dot (grown to the right). Formation of a self-avoiding random lattice starts from a seed 3PS_B_ (N_S_ = 0, where N_S_ indicates a step number) through the arrow facing of the incoming 3PS_B_ from the next step. Lattice configurations are named as (a step number, N_S_)-(configuration number from the previous step)-(configuration number at the present step). For examples, 2-3-1 and 3-34-2 indicate 1^st^ configuration of 2^nd^ step obtained from 3^rd^ configuration in 1^st^ step for 2-3-1, and 2^nd^ configuration of 3^rd^ step obtained from 3^rd^ configuration in 1^st^ step and 4^th^ configuration in 2^nd^ step for 3-34-2. All possible lattice configurations up to N_S_ = 3 are shown in Supplementary Fig. 4. In order to predict applicable numbers of self-avoiding lattices, available lattice configurations at a given N_S_ are analyzed. There are two types of available lattice configurations, *i*.*e*. an open, marked as a hollow circle and a blocked lattice configuration marked as either a half-filled (with red for left-blocked or green for right-blocked configurations) or a fully-filled circle as shown in Fig. 4a. Open, half-blocked, and full-blocked lattice configurations are easily determined by counting available numbers of arrows (binding sites for the next step) in a lattice (*i*.*e*. 2, 1, and 0 arrows in the lattices indicate open, half-, and full-blocked lattice configurations, respectively).

Overall self-avoiding random lattice configurations are represented by a pedigree chart in Fig. 4b. Although all blocked lattice configurations (up to N_S_ = 4) are fully displayed, some open configurations are skipped (indicated by dots) for clarity. Total numbers of open (Ω_O_) and blocked (full- and half-blocked) (Ω_B_) lattice configurations at N_S_ = 3 are 60 and 4 (2 and 2) among 64 available configurations $\,({{\rm{\Omega }}}_{{{\rm{N}}}_{{\rm{S}}}}={4}^{3})$. Similarly, there are 224 open and 32 blocked lattice configurations (20 full-blocked and 12 half-blocked configurations (10 happened on the left side of the lattice and 2 on the right)) out of 256 $\,({{\rm{\Omega }}}_{{{\rm{N}}}_{{\rm{S}}}}={4}^{3})$ at the 4^th^ step of lattice growth. The total number of 3PS_B_ (excluding a seed 3PS_B_) that participated in specific lattice configurations varied with (and even within) N_S_, which are indicated by magenta in the pedigree chart. Figure 4c shows a flowchart with algorithmic steps for acquiring Ω_O_ and Ω_B_ as a function of N_S_. By initially assigning the total number of trials (n_T_) and N_S_ with i and j for the unit-step increments of the trial and the step respectively, ${{\rm{\Omega }}}_{{\rm{{\rm O}}}}(\,={4}^{{{\rm{N}}}_{{\rm{S}}}}\times ({{\rm{n}}}_{{\rm{T}}}-{{\rm{n}}}_{{\rm{B}}})/{{\rm{n}}}_{{\rm{T}}}$, where n_B_ is the number of trials giving blocked lattice configurations) and ${{\rm{\Omega }}}_{{\rm{B}}}(\,={4}^{{{\rm{N}}}_{{\rm{S}}}}-{{\rm{\Omega }}}_{{\rm{O}}})$ at a given N_S_ are counted until i reaches to n_T_.

### Analysis of self-avoiding random lattice configurations

**Figure 5:**
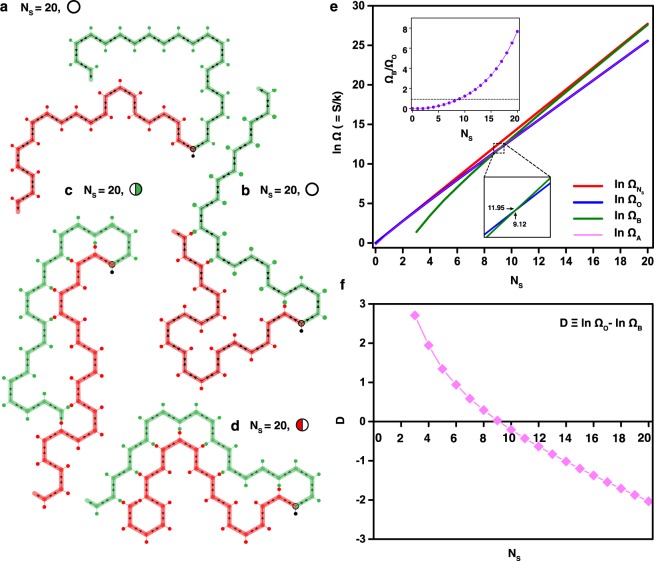
Representative lattice configurations and analysis of self-avoiding random lattice growth generated by the self-avoiding walk algorithm. **(a**–**d)** Lattice configurations of self-avoiding random growth at a N_S_ of 20. Open (**a**,**b**) and half-blocked configurations (growth blocked on either the right (**c**) or left (**d**) side of the lattices) are displayed. (**e**) Logarithmic numbers of lattice configurations ($\mathrm{ln}\,{\rm{\Omega }}\,$ = S/k, where S is entropy and k is a constant) as a function of N_S_. $\mathrm{ln}\,{\rm{\Omega }}$ obtained from the total numbers of available, open, and blocked (including half- and full-blocked) lattice configurations (${{\rm{\Omega }}}_{{{\rm{N}}}_{{\rm{S}}}}={4}^{{{\rm{N}}}_{{\rm{s}}}}$, ${{\rm{\Omega }}}_{{\rm{O}}}$, and ${{\rm{\Omega }}}_{{\rm{B}}}$, respectively) at a given N_S_ as well as from analytical evaluation of open lattice configuration (Ω_A_) are depicted. The intersection between ln Ω_O_ and ln Ω_B_ (occurred at 9.12 of N_S_) and the ratio of Ω_B_ and Ω_O_ are shown in the bottom and top insets, respectively. Ω_O_ is larger and smaller than Ω_B_ at below and above regions of the thin dotted line (marked at Ω_B_/Ω_O_ = 1 in the graph of Ω_B_/Ω_O_), respectively. (**f)** A graph of difference of $\mathrm{ln}\,{{\rm{\Omega }}}_{{\rm{O}}}$ and $\mathrm{ln}\,{{\rm{\Omega }}}_{{\rm{B}}}$ (${\rm{D}}\equiv \,\mathrm{ln}\,{{\rm{\Omega }}}_{{\rm{O}}}\mbox{--}\,\mathrm{ln}\,{{\rm{\Omega }}}_{{\rm{B}}}$) as a function of N_S_. As mentioned, D becomes 0 at N_S_ of 9.12 and the magnitude of D increases noticeably as N_S_ increases or decreases from 9.12.

Physical configurations of self-avoiding random lattices with the symbolic representations of configurations grown up to N_S_ of 20 (50 and 100) generated by the self-avoiding walk algorithm are shown in Fig. 5a–d (Supplementary Figs 5 and 6). Two-dimensional self-avoiding random lattices are self-assembled through the subsequent 3PS_B_ bindings to a seed tile of 3PS_B_, which has two binding sites, left and right leading the paths of the red and green, respectively. Here, open, half-blocked (growth blocked on either the left (a red path) or right (a green) side of the lattice), and full-blocked configurations are symbolized by a hollow, half-filled and fully-filled circle, respectively.

Figure 5e and f show logarithmic numbers of lattice configurations ($\mathrm{ln}\,{\rm{\Omega }}=S/k$, where S is entropy and k is a constant) and its difference for open and blocked self-avoiding random lattice configurations as a function of N_S_. $\mathrm{ln}\,{{\rm{\Omega }}}_{{{\rm{N}}}_{{\rm{S}}}}$, $\mathrm{ln}\,{{\rm{\Omega }}}_{{\rm{O}}}$, and $\mathrm{ln}\,{{\rm{\Omega }}}_{{\rm{B}}}$ are easily obtained from the total number of available, open, and blocked (including half-blocked and full-blocked) lattice configurations (*i*.*e*. ${{\rm{\Omega }}}_{{{\rm{N}}}_{{\rm{S}}}}={4}^{{{\rm{N}}}_{{\rm{s}}}}$, ${{\rm{\Omega }}}_{{\rm{{\rm O}}}}$, and ${{\rm{\Omega }}}_{{\rm{B}}}$) respectively at a given N_S_. In addition, the total number of open lattice configurations (Ω_A_) for a 2-dimensional hexagonal lattice model can be analytically extracted $({{\rm{\Omega }}}_{{\rm{A}}}=0.415\cdot {(\sqrt{2+\sqrt{2}})}^{2{{\rm{N}}}_{{\rm{S}}}+1}\cdot {(2{{\rm{N}}}_{{\rm{S}}}+1)}^{\frac{11}{32}})$, as shown in Fig. 5e ^24^. Although $\mathrm{ln}\,{{\rm{\Omega }}}_{{\rm{O}}}$ and $\mathrm{ln}\,{{\rm{\Omega }}}_{{\rm{A}}}$ differ by ~3% at relatively smaller N_S_, they tend to overlap completely with the difference percentage ratio $(100\times |\mathrm{ln}\,{{\rm{\Omega }}}_{{\rm{A}}}-\,\mathrm{ln}\,{{\rm{\Omega }}}_{{\rm{O}}}|/\,\mathrm{ln}\,{{\rm{\Omega }}}_{{\rm{A}}})$ of ~10^−2^% at larger N_S_. The intersection between ln Ω_O_ and ln Ω_B_ (occurred at 9.12 of N_S_) and the ratio of Ω_B_ and Ω_O_ are shown in the bottom and top insets, respectively. Ω_O_ is larger and smaller than Ω_B_ at below and above regions of the thin dotted line (marked at Ω_B_/Ω_O_ = 1 in the graph of Ω_B_/Ω_O_), respectively. In order to compare occurrences of open and blocked lattice configurations, difference (D) of ln Ω_O_ and ln Ω_B_ as a function of N_S_ are discussed (Fig. 5f). As mentioned, D becomes 0 at N_S_ of 9.12 and magnitude of D increases with increasing or decreasing N_S_ from the cross point at N_S_ = 9.12.

### Experimental observation of self-avoiding random lattices

**Figure 6:**
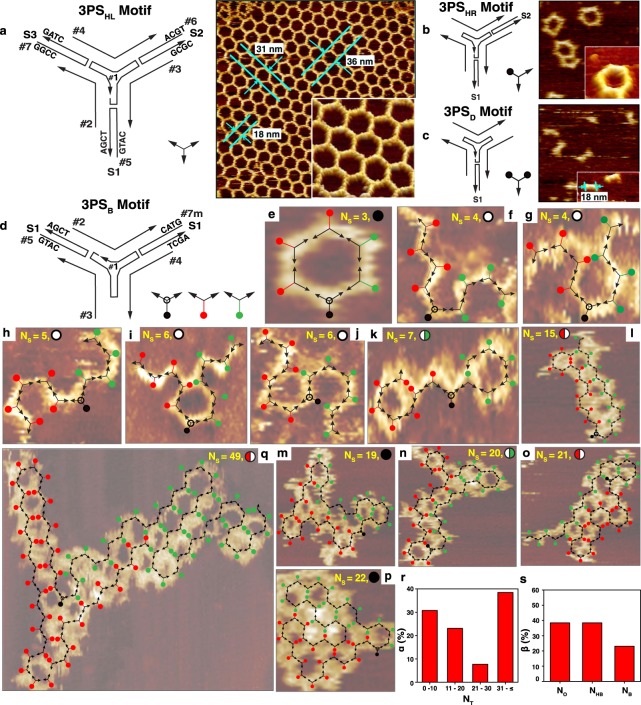
Experimental observation of self-avoiding random lattice growth with the three-point star DNA motif. (**a)** A schematic of a three-point star DNA motif (3PS_HL_) for construction of a honeycomb lattice and its representative AFM image (scan size of 500 × 500 nm^2^) of a honeycomb lattice. Seven strands constituting 3PS_HL_ are numbered as #1~#7, where palindromic self-complementary sticky-end sequences located at the end of each arm are indicated as S1, S2, and S3. A simplified 3PS_HL_ and a magnified honeycomb lattice (100 × 100 nm^2^) are shown at the right bottom corners of them. (**b)** A schematic of a three-point star DNA motif with a single blunt end (3PS_HR_) for fabrication of a hexagonal ring and its AFM image. Six strands (strand #7 removed from 3PS_HL_) and two sets (S1 and S2) of palindromic self-complementary sticky-end sequences are required. A black dot in simplified 3PS_HR_ indicates a blunt end arm. Inset in AFM image is 3-dimensional visualization of a hexagonal ring. (**c)** A schematic of a three-point star DNA motif with double blunt ends (3PS_D_) for formation of a 3PS dimer and its AFM image. Five strands (#6 and #7 removed from 3PS_HL_) and single set (S1) of palindromic self-complementary sticky-end sequences is required. Inset in AFM image is 3-dimensional visualization of 3PS dimers. (**d)** A schematic of a three-point star DNA motif with a blunt end (3PS_B_) for demonstration of a self-avoiding random lattice. Strand #6 is removed from 3PS_HL_ and self-complementary sticky-end sequences in #7 are modified. A blunt-end in a simplified 3PS_B_ is marked with a black (served as a seed), a red (grown to the left), or a green dot (grown to the right) in order to easily analyze the lattice configurations. (**e**–**q)** Representative AFM images of self-avoiding random lattices comprised of 3PS_B_. Either an open, a half-blocked or a full-blocked lattice configuration at a given step number is indicated in each image. In order to clarify the growth visualization of lattice configurations, simplified 3PS_B_ are overlaid on AFM images. (**r)** A plot of percentage of total number of 3PS_B_ motifs (α) in that specific range, *i*.*e*. below 10, 11–20, 21–30, and above 30. (**s)** A bar graph of percentages of the total number of open, half-blocked, and a full-blocked lattice configurations (β).

Three different DNA nanostructures (a honeycomb lattice, a hexagonal ring, and a three-point star dimer) are constructed by slightly modified three-point star DNA motifs in order to test their applicability in the growth of self-avoiding random lattices (See Fig. 6, Supplementary Fig. 7, and Supplementary Table 5). Figure 6a shows a schematic of a three-point star DNA motif (3PS_HL_) for construction of a honeycomb lattice (a simplified one shown at a right bottom) and its representative AFM image of a honeycomb lattice. A 3PS_HL_ is comprised of 7 strands (marked as #1~#7) with palindromic self-complementary sticky-end sequences (indicated as S1, S2, and S3) located at the end of each arm^41,42^. Schematics and representative AFM images of three-point star DNA motifs with a single (3PS_HR_, for fabrication of a hexagonal ring) and double blunt ends (3PS_D_, for formation of a 3PS dimer) are shown in Fig. 6b and c. A 3PS_HR_ (a black dot in simplified 3PS_HR_ indicates a blunt end arm as shown in Fig. 6b) and a 3PS_D_ (two black dots in simplified 3PS_D_ represent the blunt end arms in Fig. 6c) need 6 strands (strand #7 removed from 3PS_HL_) with two sets (S1 and S2) of palindromic self-complementary sticky-end sequences, and 5 strands (#6 and #7 removed from 3PS_HL_) with a single set (S1) of palindromic self-complementary sticky-end sequences, respectively. From the observation of the AFM images, honeycomb lattices, hexagonal rings, and 3PS dimers are well formed in agreement with the design schemes with relatively higher production yields than cross-tile lattices made of four-point star motifs^43^.

Figure 6d–s show the representative experimental results and analysis of self-avoiding random lattices grown by the 3PS DNA motifs (3PS_B_). In 3PS_B_, a #6 strand from 3PS_HL_ is removed and self-complementary sticky-end sequences in #7 are replaced from S3 to S1. A blunt-end in a simplified 3PS_B_ shown in the right bottom of Fig. 6d is marked with either a black (served as a seed), a red (grown to the left), or a green dot (grown to the right) in order to easily evaluate the lattice configurations. Representative AFM images with the lattice configurations (either an open, a half-blocked or a full-blocked configuration at a given step number) of self-avoiding random lattices comprised of 3PS_B_ are displayed in Fig. 6e–p. Simplified 3PS_B_ motifs are overlaid on AFM images to enhance the visibility of lattice configurations. Figure 6r,s display percentages of the total number of 3PS_B_ motifs (α) in specific ranges (*i*.*e*. below 10, 11–20, 21–30, and above 30) and percentages of total number of open, half-blocked and full-blocked lattice configurations (β) obtained from the AFM data. Although it would be difficult to form relatively larger self-avoiding lattices due to the existence of a blunt end in a 3PS_B,_ as we anticipated, interestingly we observe that lattices having more than 31 numbers of 3PS_B_ are dominant (38.5% among all evaluated lattices). In addition, the percentages of lattice configurations in the range of 3 to 49 of N_S_ are examined. Open (blocked) lattice configurations are dominant below (above) N_S_ = 9.12, which agree well with the simulation results discussed in Fig. 5e,f.

## Discussion

We discuss methodologies to calculate the numerical value of π and to evaluate a possible number of self-avoiding walk paths with the aid of computational MC simulation. Additionally, we demonstrate the calculation of π and evaluation of applicable self-avoiding walk paths by distinct DNA nanostructures. Finally, we analyze the trend of numerical variations of π as functions of DNA concentration and the total number of trials for π calculation, and the behaviour of self-avoiding random DNA lattice growth evaluated through number of growth steps for the self-avoiding walk path. From observation of experimental calculations of π (π_exp_) demonstrated by constructing two different types of DNA nanostructures (*i*.*e*. double crossover DNA lattices and DNA rings), fluctuation of π_exp_ from known π tends to decrease as either DNA concentration or the number of trials increases. Based upon experimental observation of self-avoiding random lattices grown by the three-point star DNA motifs, the percentage of lattice configurations is examined. Open (blocked) lattice configurations are dominant below (above) the step number of 9.12 (at this step number obtained by simulation, numbers of open and blocked configurations are the same). This in depth study of numerical calculation of mathematical constants and characteristic estimation of abstract models via DNA provides a novel perspective for the applicability of DNA in the field of science and engineering.

## Methods

### DNA nanostructure fabrication

Synthetic oligonucleotides purified *via* high-performance liquid chromatography were purchased from Bioneer (Daejeon, Korea). Double-crossover (DX) DNA lattices were formed by the 2-step free solution annealing method. First, individual strands of either DX (without hairpins, PR0 and PS0) or DXH (with hairpins, PR1 and PS1) motif were mixed with equimolar concentration (800 nM) in 1 × TAE/Mg^2+^ buffer solution (40 mM Tris, 20 mM Acetic acid, 1 mM EDTA (pH 8.0), and 12.5 mM magnesium acetate). These strand mixtures of each motif (*i*.*e*. PR0, PS0, PR1, and PS1) in the test tubes were then slowly cooled from 95 to 25 °C by placing them in a Styrofoam box containing 2 L of boiled water for about 2 days to facilitate hybridization. In succession, an appropriate amount of each motif was added into a new test tube to obtain DXH_0_ DNA lattices (final concentrations of individual motifs were [PR0] = [PS0] = 100 nM, and [PR1] = [PS1] = 0 nM). Similarly, sets of motif concentrations ([PR0], [PS0], [PR1], and [PS1] = 75, 100, 25, and 0 nM; 50, 100, 50, and 0 nM; 25, 100, 75, and 0 nM; 50, 50, 50, and 50 nM; 0, 50, 100, and 50 nM; 0, 0, 100, and 100 nM) were prepared to construct DXH_0.25_, DXH_0.5_, DXH_0.75_, DXH_1.0_, DXH_1.5_, and DXH_2.0_ DNA lattices, respectively. Second step annealing was performed by placing sample test tubes in a Styrofoam box containing 2 L of water (initial temperature, 40 °C) and cooling them from 40 °C to 25 °C for about 24 hours to obtain DX DNA lattices. (Fig. 2, Supplementary Fig. 2, Supplementary Tables 1 and 2)

DNA rings were formed by mixing a stoichiometric quantity of each strand in a buffer containing a mica substrate (size of 5 × 5 mm^2^). This strand mixture with mica was annealed in a test tube by slowly cooling from 95 to 25 °C in a Styrofoam box. Eventually, DNA rings formed on the mica surface with different coverages depending upon the concentration of a T motif. DNA rings with a five different T motif concentrations of 2, 5, 8, 10 and 20 nM were prepared and analyzed. (Fig. 2, Supplementary Fig. 3, Supplementary Tables 3 and 4)

Honeycomb lattices, hexagonal rings, 3PS dimers, as well as self-avoiding random lattices were constructed by specific three-point star motifs; 3PS_HL_, 3PS_HR_, 3PS_D_, and 3PS_B_ motifs. They were formed by mixing stoichiometric quantities of each strand in the buffer by cooling from 95 °C to 25 °C in a Styrofoam box. Final concentrations of 3PS for all DNA nanostructure configurations were 200 nM. (Fig. 6, Supplementary Fig. 7, Supplementary Table 5)

### AFM imaging

5 μL of DNA nanostructures (*i*.*e*. DX lattices, honeycomb lattices, hexagonal rings, 3PS dimers, and self-avoiding random lattices) in buffer solution prepared *via* the free-solution annealing method were dropped on a freshly cleaved mica surface. A 30 μL of 1 × TAE/Mg^2+^ buffer solution was then placed onto the mica, and another 20 μL was placed onto the silicon nitride AFM tip (NP-S10, Veeco Inc., CA, USA). To image DNA rings fabricated through the MAG method, a mica substrate with preformed DNA rings was taken from a test tube and placed on a metal puck. Then, 30 μL of buffer was pipetted onto the mica substrate, and another 20 μL was dispensed onto an AFM tip. Corresponding AFM images were then obtained using a Multimode Nanoscope (Veeco Inc., CA, USA) in the fluid-tapping mode (Figs 2 and 6).

## Supplementary information

SUPPLEMENTARY INFO
