# Supplementary material for: Calculation of π and Classification of Self-avoiding Lattices via DNA Configuration
Source: Sci Rep. 2019 Feb 19;9:2252. doi: 10.1038/s41598-019-38699-0 (PMC6381155; doi:10.1038/s41598-019-38699-0)
Supplement: Supplementary file 1 — SUPPLEMENTARY INFO [file 41598_2019_38699_MOESM1_ESM.pdf]

Supplementary Information

Calculation of π and Classification of Self-avoiding Lattices via DNA Configuration

Anshula Tandon[1#], Seungjae Kim[1#], Yongwoo Song[1], Hyunjae Cho[1],
Saima Bashar[1], Jihoon Shin[2], Tai Hwan Ha[2,3]* & Sung Ha Park[1]*

[1] Department of Physics and Sungkyunkwan Advanced Institute of Nanotechnology (SAINT), Sungkyunkwan University, Suwon 16419, Korea

[2] Hazards Monitoring BNT Research Center,
Korea Research Institute of Bioscience and Biotechnology (KRIBB),
Daejeon 34141, Korea

[3] Department of Nanobiotechnology, KRIBB School of Biotechnology,
Korea University of Science and Technology (UST), Daejeon 34113, Korea

[#] These authors contributed equally to this work

* Correspondence and requests for materials should be addressed to T.H.H. (email: taihwan@kribb.re.kr) or S.H.P. (email: sunghapark@skku.edu)

Supplementary Information Contents

Supplementary Figures 1 ~ 7 and

Supplementary Tables 1 ~ 5.

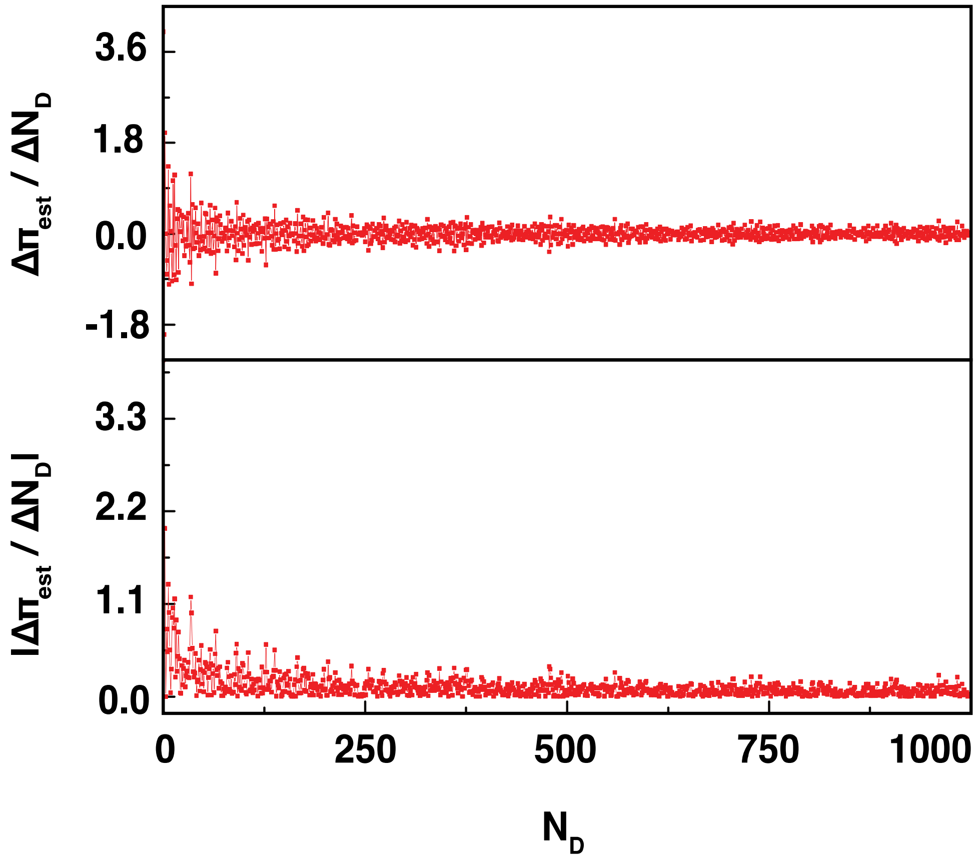

Supplementary Figure 1. Differentiation of π_{est} per unit number of dots as a function of N_D using the computer simulation. Differentiation of π_{est} per unit number of dots (= $\Delta\pi_{est}/\Delta N_D$) and its absolute value (= $|\Delta\pi_{est}/\Delta N_D|$) as a function of N_D are obtained from Figure 1e (π_{est} as a function of N_D). Differences of the π_{est} per unit number of dots tend to decrease with increase in N_D because π_{est} at larger N_D has more chance to give an accurate value of π_{known} through the simulation. (Figure 1)

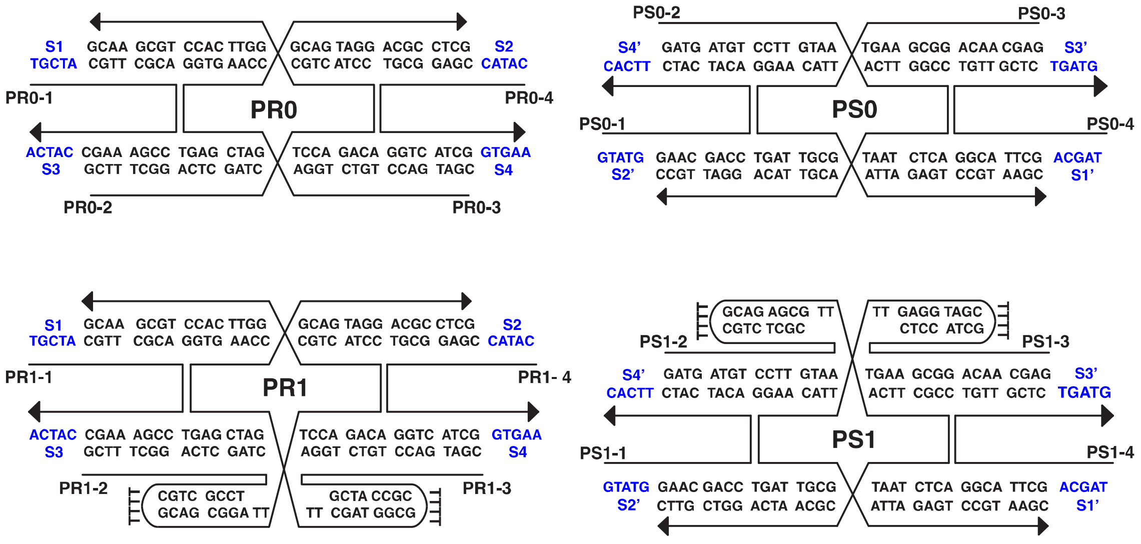

Supplementary Figure 2. Schematic illustration of double-crossover without (DX) and with hairpins (DXH) motifs having their base sequences for formation of DNA lattices. Schematics of DX (PR0, PS0) and DXH (PR1, PS1) motifs with their respective strands. The complementary set (S# and S#') of sticky-end sequences are indicated in blue. (Figure 2 and Figure 3)

Strand name	Total number of bases	Sequence (5′ to 3′)
PR0-1	26	**TGCTA** CGTT CGCA CCGA AAGC **CATCA**
PR0-2	48	GCTT TCGG ACTC GATC TCCA GACA CCTA CTGC GGTT CACC TGCG AACG
PR0-3	48	CGAT GACC TGTC TGGA GATC GAGT GGTG AACC GCAG TAGG ACGC CTCG
PR0-4	26	**CATAC** CGAG GCGT GGTC ATCG **GTGAA**
PR1-2	70	GCTT TCGG ACTC GATC TCCG CTGC TTTT GCAG CGGA TTTC CAGA CACC TACT GGGG TTCA CCTG CGAA CG
PR1-3	70	CGAT GACC TGTC TGGA GCTA CCGC TTTT GCGG TAGC TTGA TCGA GTGG TGAA CCGC AGTA GGAC GCCT CG
PS0-1	26	**GTATG** GAAC GACC ACAT CATC **TTCAC**
PS0-2	48	GATG ATGT CCTT GTAA ACTT CGCC ACTC TAAT CGCA ATCA GGTC GTTC
PS0-3	48	GAGC AACA GGCG AAGT TTAC AAGG TGAT TGCG ATTA GAGT CCGT AAGC
PS0-4	26	**TAGCA** GCTT ACGG TGTT GCTC **TGATG**
PS1-2	70	GATG ATGT CCTT GTAA CGCT CTGC TTTT GCAG AGCG TTAC TTCG CCAC TCTA ATCG CAAT CAGG TCGT TC
PS1-3	70	GAGC AACA GGCG AAGT CTCC ATCG TTTT CGAT GGAG TTTT ACAA GGTG ATTG CGAT TAGA GTCC GTAA GC

Supplementary Table 1. Strand details representing number of bases and DNA sequence of each strand of DX and DXH motifs.

Table of Sticky ends			
	5’ to 3’	3’ to 5’	
S1	TGCTA	ACGAT	S1’
S2	CATAC	GTATG	S2’
S3	CATCA	GTAGT	S3’
S4	GTGAA	CACTT	S4’

Supplementary Table 2. Sequence of sticky-ends used in DX and DXH motifs.

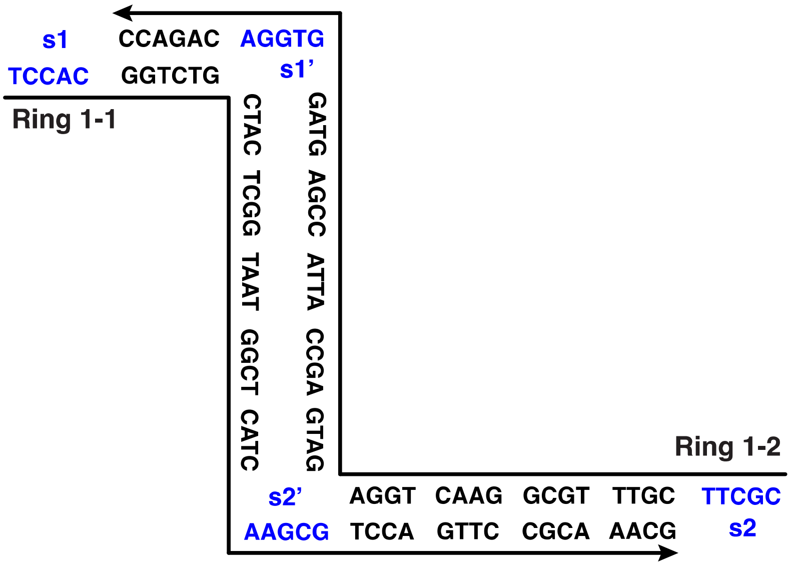

Supplementary Figure 3. Schematic of a T motif showing base sequences for construction of DNA rings. A T motif consists of two strands (*i.e.* Ring 1-1 and Ring 1-2) and the complementary sticky-end sets of the T motif are indicated as s# and s#’ in blue. (Figure 2 and Figure 3)

Strand name	Total number of bases	Sequence (5′ to 3′)
Ring 1-1	52	**TCCAC** GGTCTG CTAC TCGG TAAT GGCT CATC **AAGCG** TCCA GTTC CGCA AACG
Ring 1-2	52	**CGCTT** CGTT TGCG GAAC TGGA GATG AGCC ATTA CCGA GTAG **GTGGA** CAGACC

Supplementary Table 3. Strand details representing the number of bases and DNA sequence of each strand of a T motif.

**Table of Sticky ends**			
	**5′ to 3′**	**3′ to 5′**	
**s1**	**TCCAC**	**AGGTG**	**s1’**
**s2**	**CGCTT**	**GCGAA**	**s2’**

Supplementary Table 4. Sequence of sticky-ends used in a T motif.

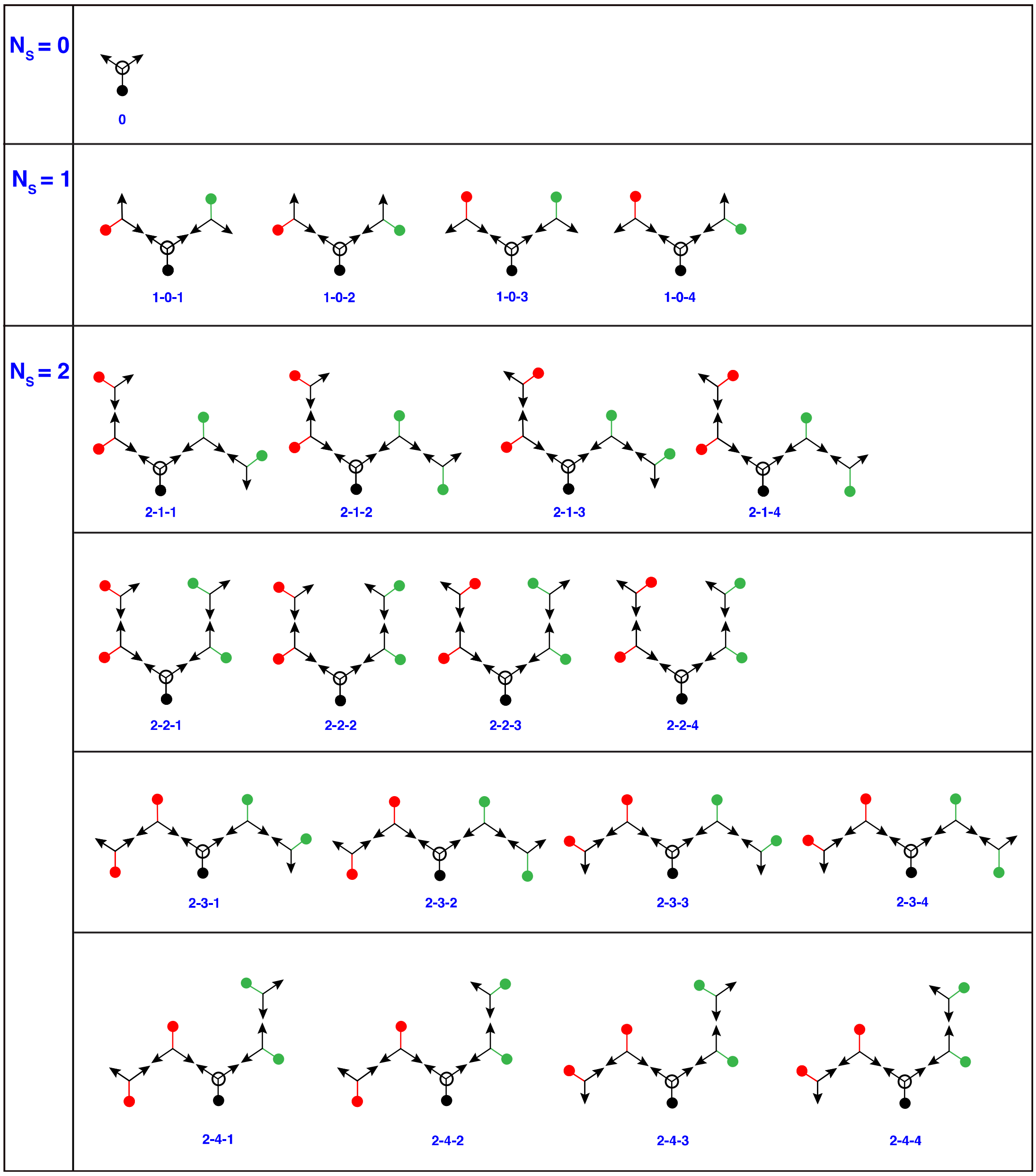
$N_S = 0$
0
$N_S = 1$
1-0-1
1-0-2
1-0-3
1-0-4
$N_S = 2$
2-1-1
2-1-2
2-1-3
2-1-4
2-2-1
2-2-2
2-2-3
2-2-4
2-3-1
2-3-2
2-3-3
2-3-4
2-4-1
2-4-2
2-4-3
2-4-4

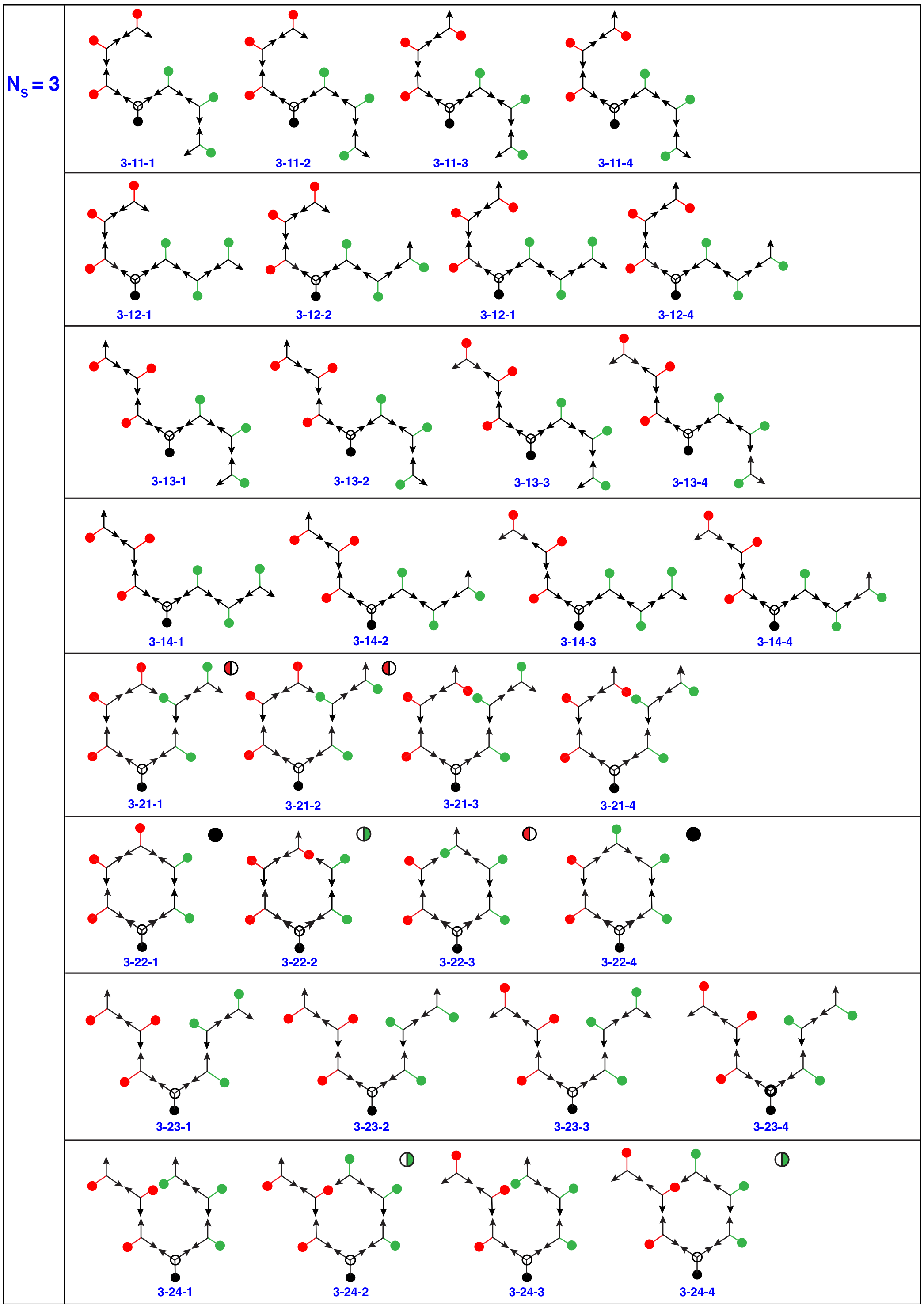
$N_S = 3$
3-11-1
3-11-2
3-11-3
3-11-4
3-12-1
3-12-2
3-12-1
3-12-4
3-13-1
3-13-2
3-13-3
3-13-4
3-14-1
3-14-2
3-14-3
3-14-4
3-21-1
3-21-2
3-21-3
3-21-4
3-22-1
3-22-2
3-22-3
3-22-4
3-23-1
3-23-2
3-23-3
3-23-4
3-24-1
3-24-2
3-24-3
3-24-4

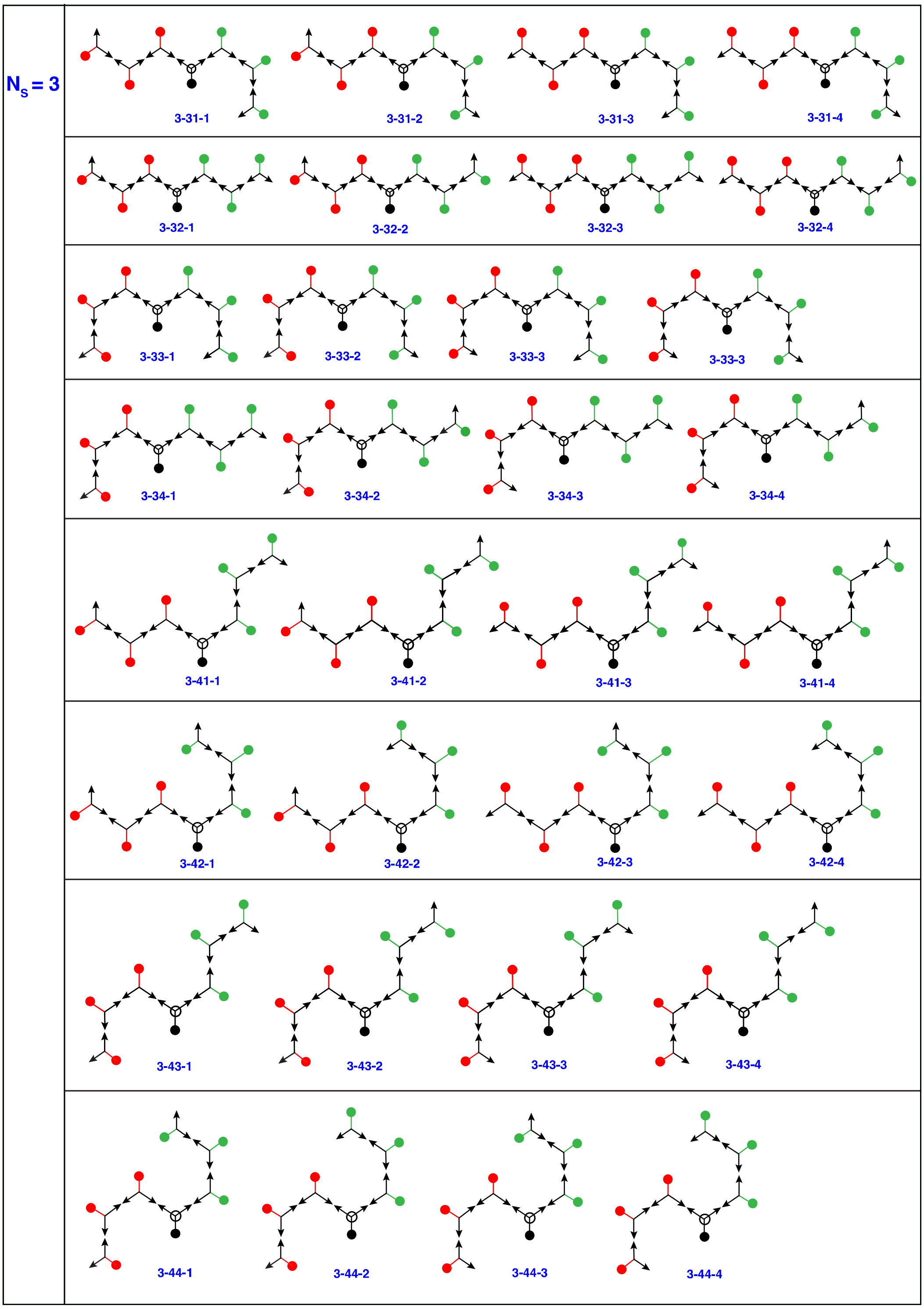

Supplementary Figure 4. Lattice configuration of self-avoiding random lattice growth demonstrated with the three-point star motif having a blunt-end. Schematic representations of all possible lattice configurations (up to step number, N_S = 3) constructed by a three-point star motif having a blunt-end ($3PS_B$). A blunt-end is marked with either a black (served as a seed), a red (grown to the left), or a green dot (grown to the right). Lattice configurations are named as (step number, N_S)-(configuration number from the previous step)-(configuration number at the present step). For instance, 3-34-2 represents 2nd configuration of 3rd step obtained from 3rd configuration in 1st step and 4th configuration in 2nd step. (Figure 4)

$N_S = 50$, ◯

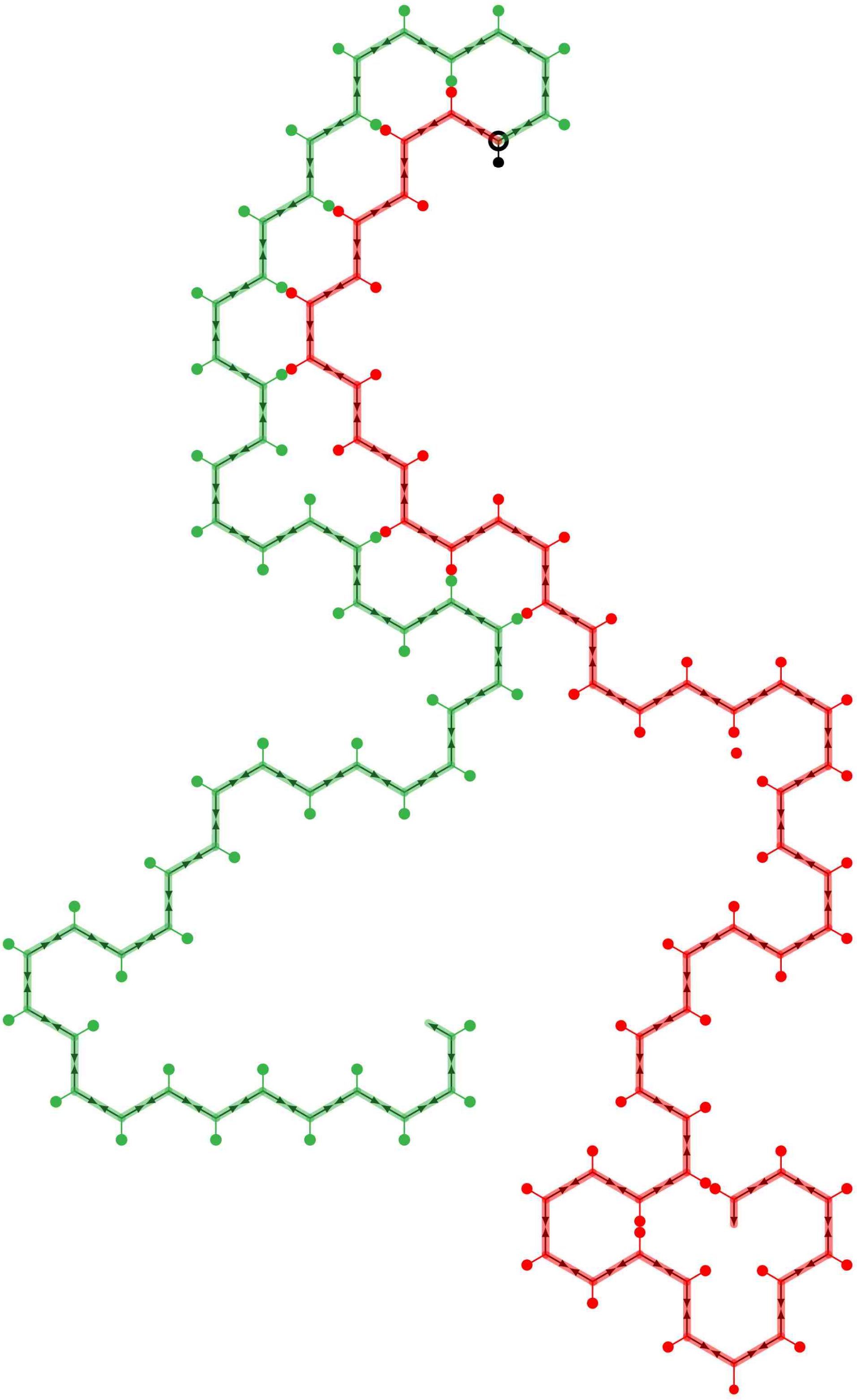

$N_S = 50$, ○

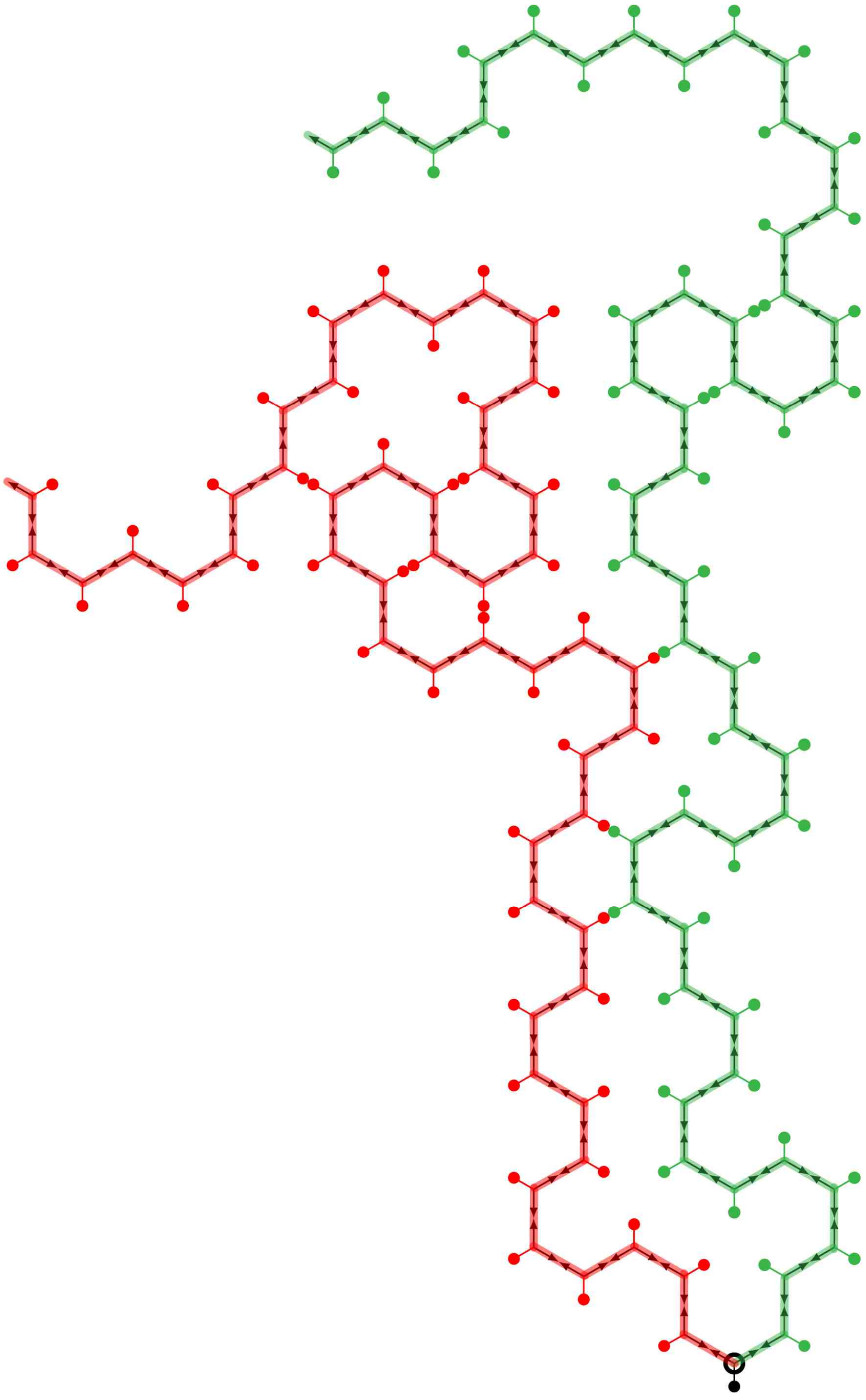

$N_S = 50$,

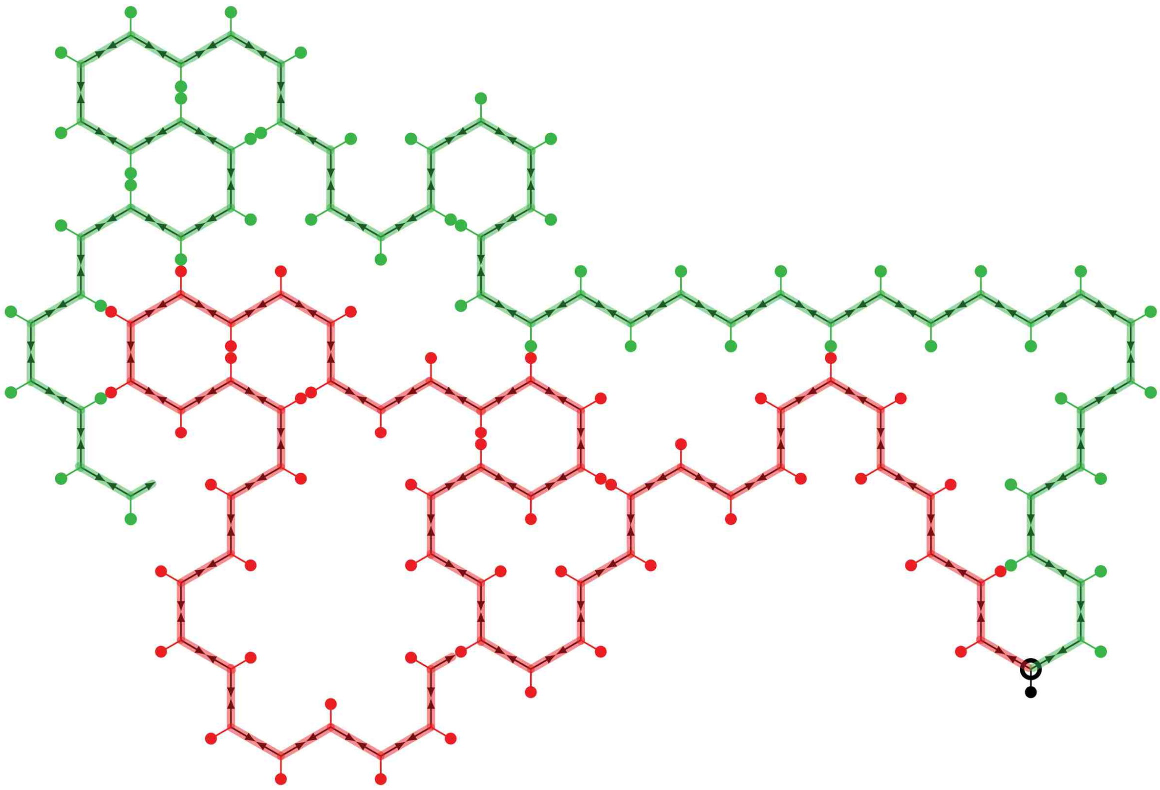

Supplementary Figure 5. Three representative self-avoiding random lattice configurations at a step number, $N_S = 50$ generated by the self-avoiding walk algorithm. Open, open, and half-blocked (growth blocked on the left side of the lattices) configurations are displayed, respectively. (Figure 5)

$N_S = 100$, ◯

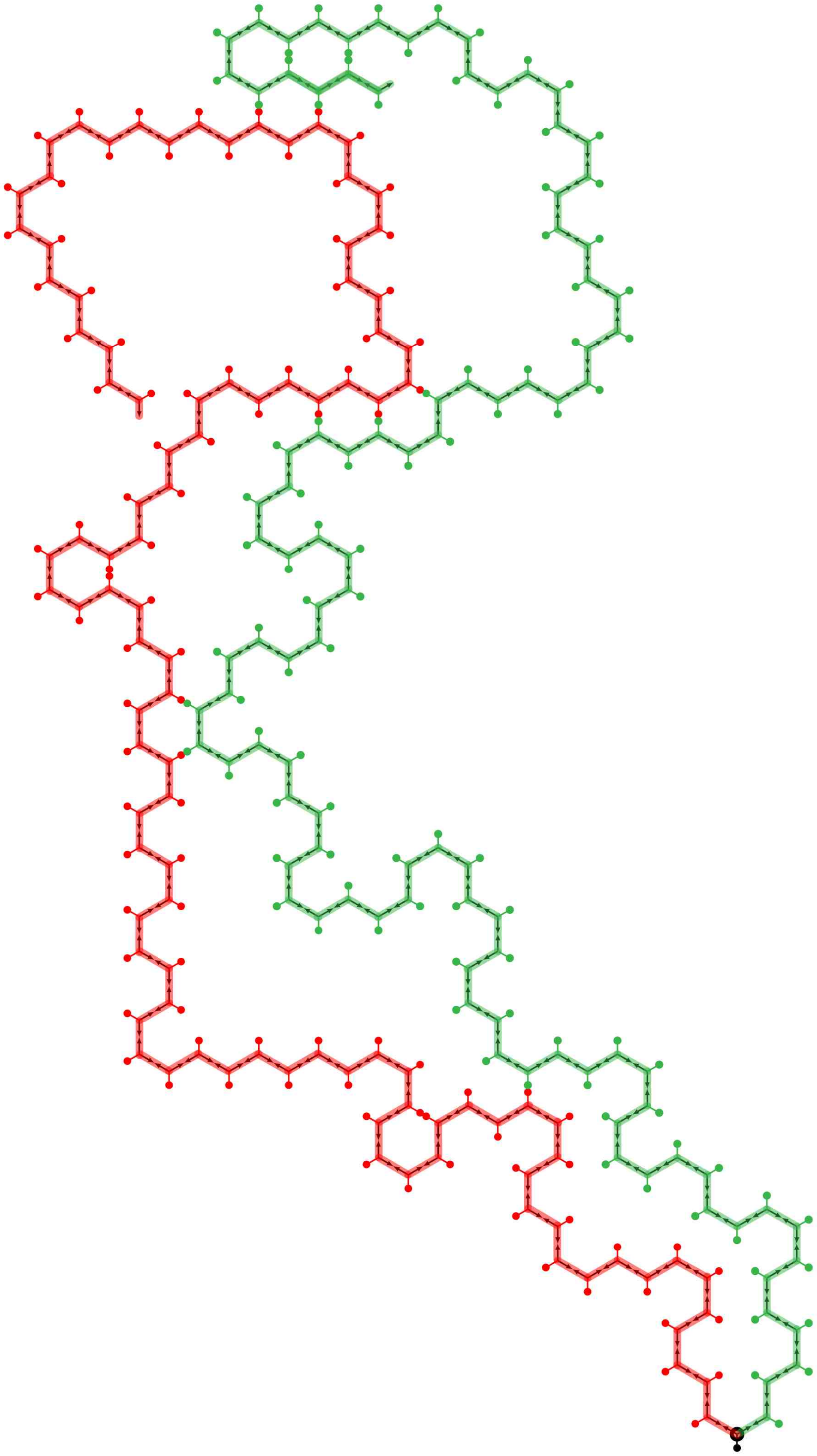

N_s = 100, ◯

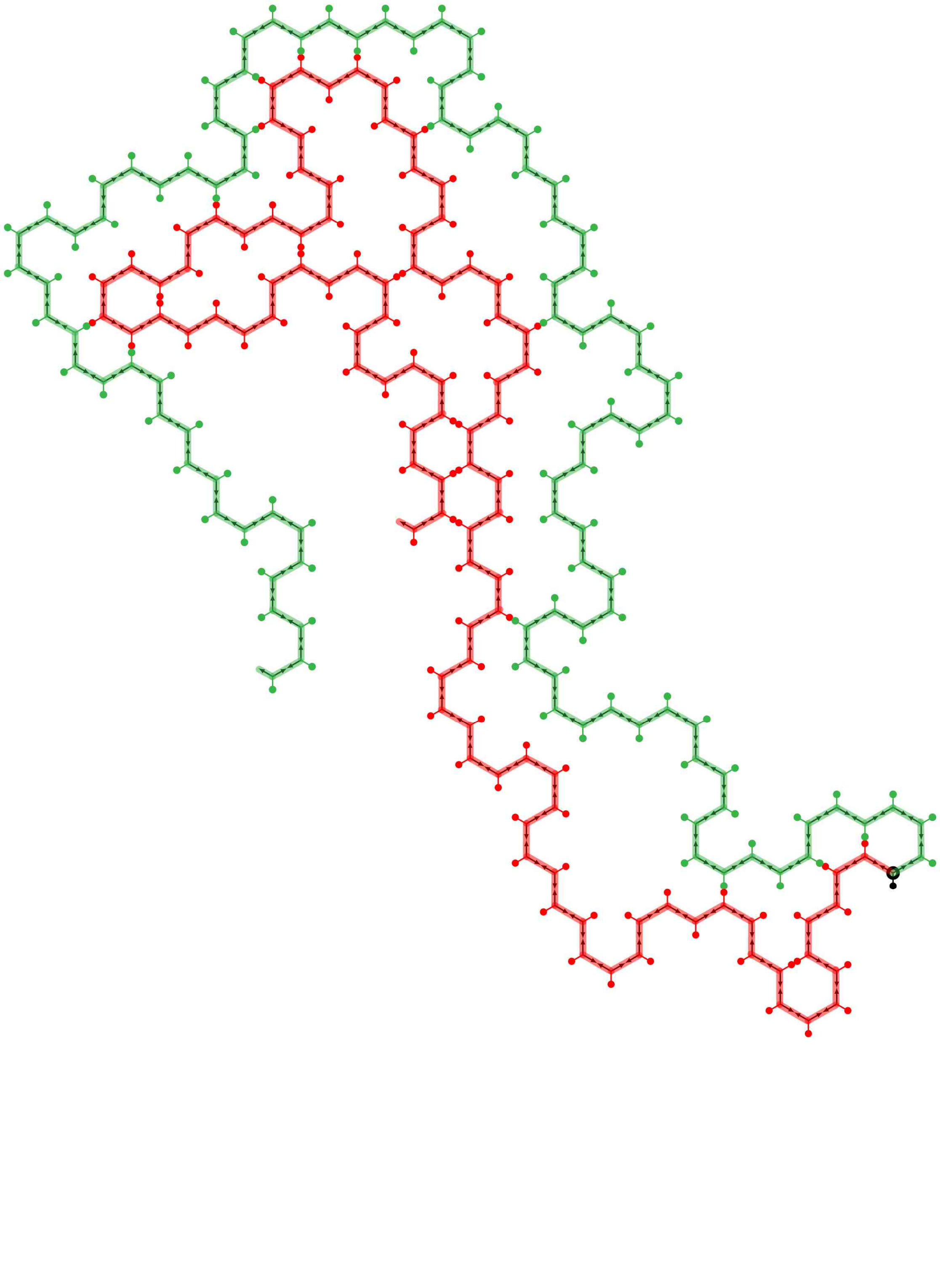

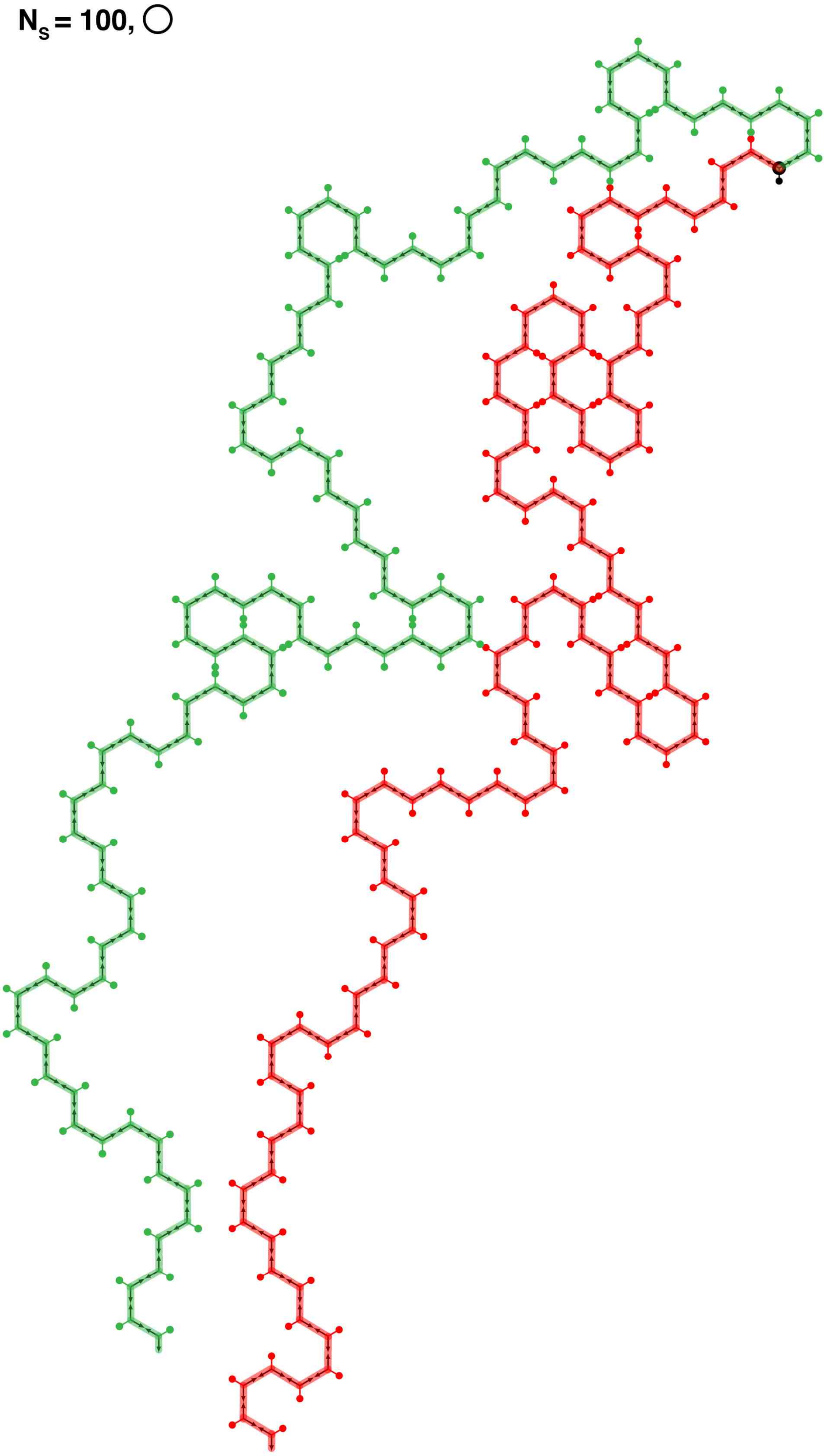

N_S = 100, ◯

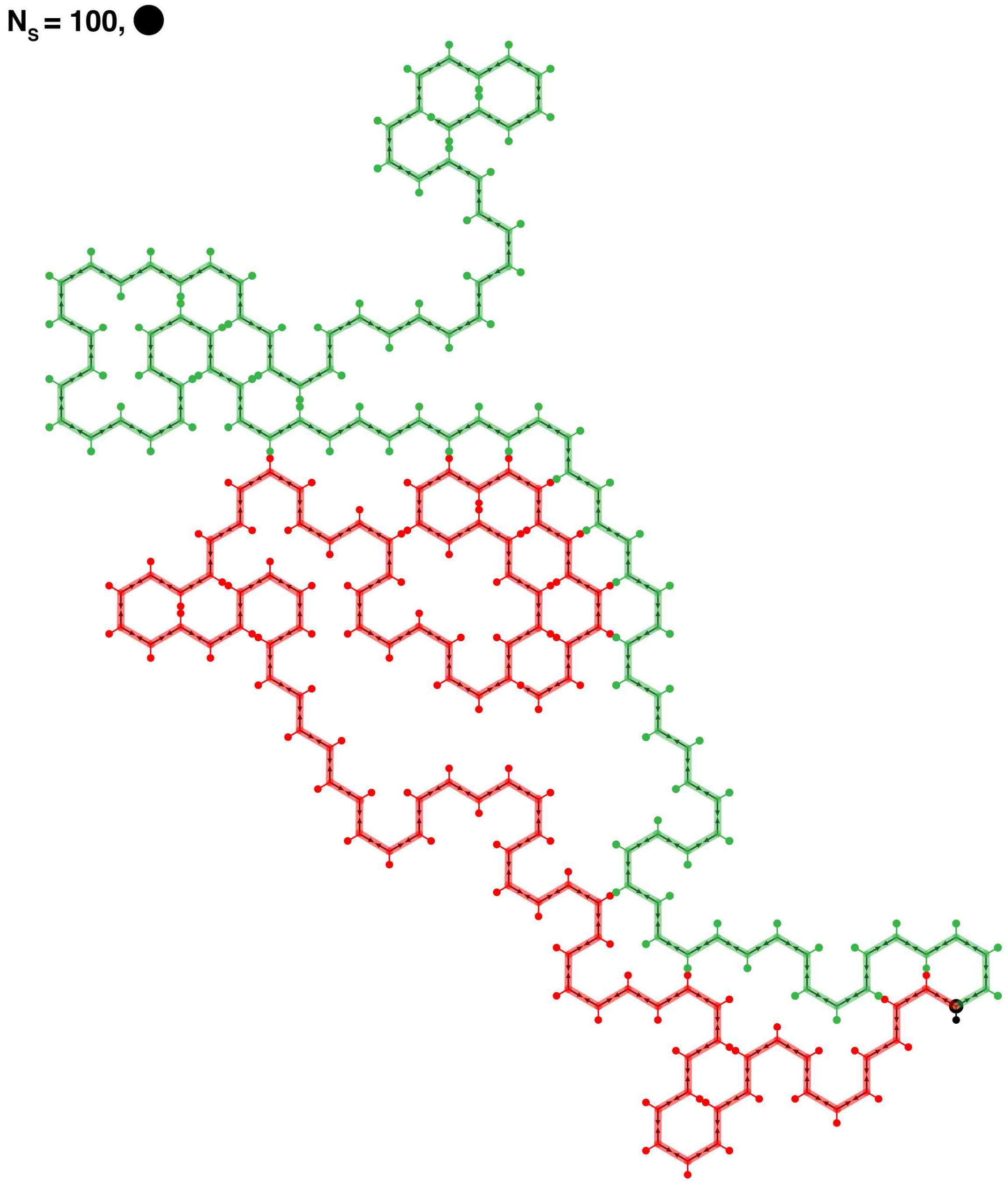

Supplementary Figure 6. Four representative self-avoiding random lattice configurations at a step number, $N_S = 100$ generated by the self-avoiding walk simulation. Open, open, open, and full-blocked (growth blocked on both side of the lattices) configurations are displayed, respectively. (Figure 5)

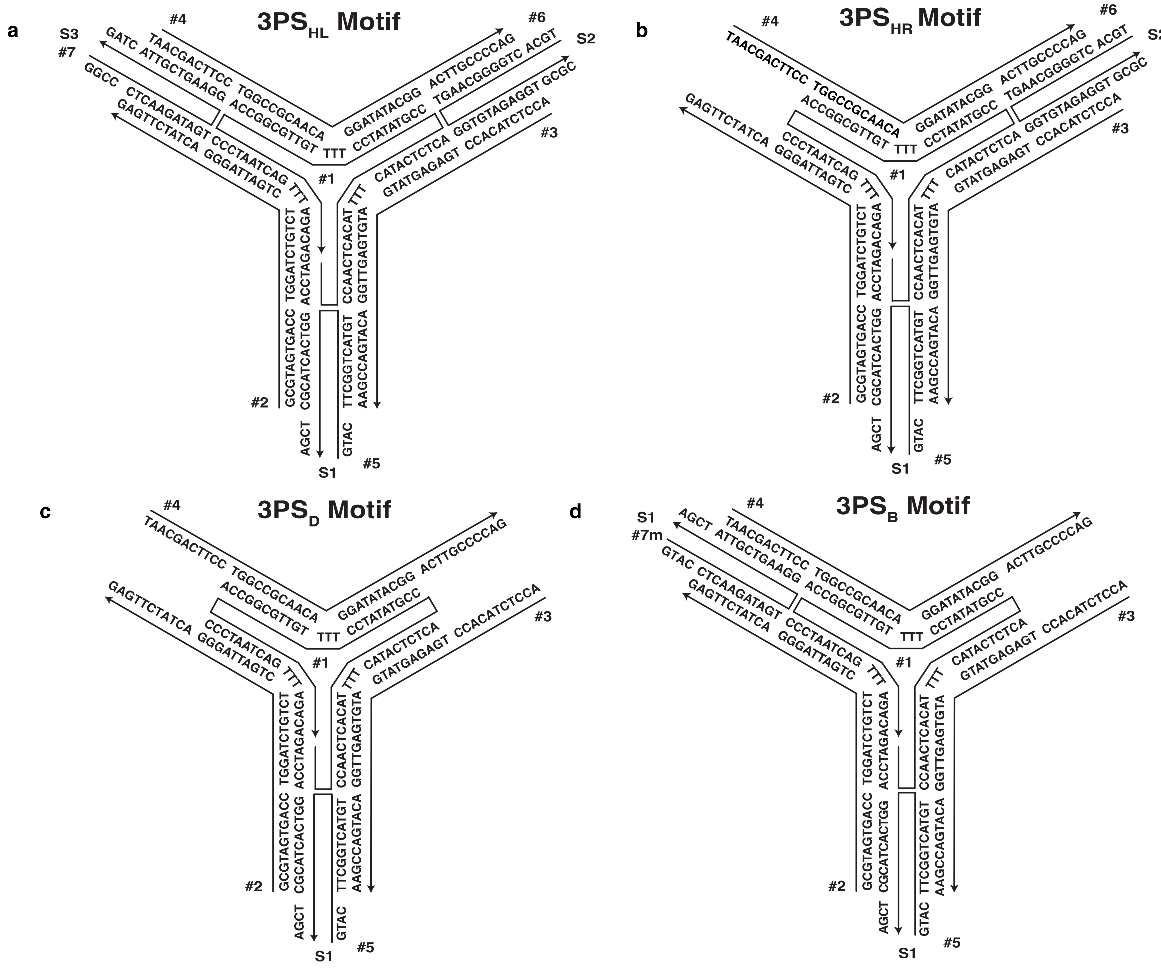

Supplementary Figure 7. Schematics of four different types of three point star motifs (3PS). a-d, Schematic sequence diagrams of three-point star DNA motifs, $3PS_{HL}$, $3PS_{HR}$, $3PS_{D}$, and $3PS_{B}$ for constructions of a honeycomb lattice, a hexagonal ring, a 3PS dimer, and a self-avoiding random lattice, respectively. a, Seven strands constituting $3PS_{HL}$ are numbered as #1 ~ #7, where palindromic self-complementary sticky-end sequences located at the end of each arm are indicated as S1, S2, and S3. b, Six strands (strand #7 removed from $3PS_{HL}$) and two sets (S1 and S2) of palindromic self-complementary sticky-end sequences are required for $3PS_{HR}$. c, $3PS_{D}$ consists of five strands (#6 and #7 removed from $3PS_{HL}$) and single set (S1) of palindromic self-complementary sticky-end sequences are required for $3PS_{D}$. d, For $3PS_{B}$, strand #6 is removed from $3PS_{HL}$ and self-complementary sticky-end sequences in #7 are modified. (Figure 6)

Strand name	Total number of bases	Sequence (5′ to 3′)
#1	72	GATCCA CCAACT CACAT TTT CATACTCTCA CCGTATATCC TTT TGTTGCGGCCA CCCTAATCAG TTT AGACA
#2	43	GCGTAGTGACC TGGATCTGTCT CTGATTAGGG ACTATCTTGAG
#3	43	ACCTCTACACC TGAGAGTATG ATGTGAGTTGG ACATGACCGAA
#4	43	TAACGACTTCC TGGCCGCAACA GGATATACGG ACTTGCCCCAG
#5	30	**GTAC** TTCGGTCATGT GGTCACTACGC **TCGA**
#6	30	**TGCA** CTGGGGCAAGT GGTGTAGAGGT **GCGC**
#7	30	**GGCC** CTCAAGATAGT GGAAGTCGTTA **CTAG**
#7m	30	**GTAC** CTCAAGATAGT GGAAGTCGTTA **TCGA**

Supplementary Table 5. Strand details representing number of bases and DNA sequence of each strand of 3PS motifs. Sequences of self-complementary palindromic sticky-ends are marked in bold.
